# The Oncology Care Model for Advanced Practitioners

**Published:** 2017-09-01

**Authors:** Wendy H. Vogel

**Affiliations:** Wellmont Cancer Institute, Kingsport, Tennessee

The Centers for Medicare & Medicaid Services (CMS) Oncology Care Model (OCM) is a new, multipayer model that focuses on higher quality, coordinated oncology care (CMS, 2016a). The OCM will be undertaken for a 5-year period from July 1, 2016, to June 30, 2021. Practices and payers applied for participation and nearly 200 practices and 17 commercial payers were selected (CMS, 2016b). The chosen practices involved committed to provide enhanced services (for example, care coordination and navigation) to Medicare beneficiaries and to use national treatment guidelines (such as the National Comprehensive Cancer Network and the American Society of Clinical Oncology Guidelines). Chosen commercial insurers will align their oncology payment models with Medicare’s model. 

The rationale for this care/payment model is to attempt to provide a higher quality of care and lower costs for those over 65 years of age with cancer. This model is part of the Affordable Care Act (ACA) approach called "Better, Smarter, Healthier" in an effort to reward quality of care, not quantity of care (US Department of Health and Human Services, 2015). Stated goals of this model are better care, smarter spending, and healthier people. 

## PAYMENT SYSTEM

Practices in the OCM will receive an incentive for comprehensively and appropriately addressing the complex care needs of individuals undergoing chemotherapy and to provide services that improve patient experiences and health outcomes. The payment system is a two-part distribution system. 

The first part is a per-beneficiary monthly enhanced oncology services payment of $160 for the duration of the episode of care (a 6-month period that may begin at the launch of the OCM [July 1, 2016] or at the initiation of chemotherapy). 

The second part of the payment system is the potential for performance-based incentive payments for care. Performance-based payments will occur in 6-month intervals and are based on financial savings compared with a risk-adjusted target amount and on a scoring of quality measures. One can view how these payments are determined and how performance quality measures are calculated at innovation.cms.gov/initiatives/oncology-care.

There are 12 quality measures grouped into 4 categories: (1) communication and care coordination; (2) person- and caregiver-centered experience and outcomes; (3) clinical quality of care; and (4) patient safety. They will be measured using claims information, patient/caregiver surveys, and practice-reported registry information. For example, claims will be examined for hospital or emergency department admissions. Another example is that practices will report depression and pain assessments and how they were managed. 

Patients (beneficiaries) will be notified that their oncology practice is participating in the OCM. (This letter template can be viewed at innovation.cms.gov/Files/x/ocm-beneletter.pdf.) As part of the quality assessment, patients will be surveyed by mail about their perception of care quality, their understanding of treatment benefits and risks, and their inclusion in decision-making. They will be surveyed on whether they were screened for depression or pain and whether they are satisfied with how it was managed. Other survey questions include how thoroughly and promptly questions were answered or test results given. 

## BENEFITS AND REQUIREMENTS

Payers participating in the OCM hope to benefit from savings, better outcomes for their beneficiaries, and greater information on care quality (Thomas & Ward, 2016). Payers may utilize this model to design their own financial incentives to support their beneficiaries. They will be able to examine the cost and quality of care provided by physician practices. The main incentive for payers is to reduce overall expenditures on cancer care by incorporating value-based payment. 

The practices participating in the OCM will have to undergo significant structural changes (Thomas & Ward, 2016), including but not limited to electronic health record reporting, increasing access to clinicians, and increasing patient navigation. Practices must provide 24 hours a day, 7 days a week patient access to clinicians, with real-time access to the practice’s medical records. Electronic health record systems must be Office of the National Coordinator for Health Information Technology–certified and achieve stage 2 meaningful use by the end of the third OCM year. An example of stage 2 meaningful use according to the CMS is the use of secure electronic messaging to communicate with patients (CMS, 2012). Navigator programs must be expanded to include not only care coordination but also patient education, transportation services if needed, financial counseling, support referrals, community outreach, and access to clinical trials. Patient encounter documentation must be reported; it includes the 13 components of the Institutes of Medicine Care Management Plan, such as treatment goals, expected response to treatment, advanced care planning, treatment cost estimates, psychosocial need planning, and survivorship planning. In addition, the rationale for therapies should be documented as well as which clinical decision-making tools/guidelines are used. Data obtained from the OCM will be utilized to further continuous quality improvement (Thomas & Ward, 2016).

Oncology advanced practitioners are part of the team caring for patients under the OCM. All members of the team must maintain up-to-date knowledge of OCM components. It will take the entire multidisciplinary team to meet the quality measures and cost-saving expectations.
